# Comparison of Inhibitory Effects of Safflower Decoction and Safflower Injection on Protein and mRNA Expressions of iNOS and IL-1*β* in LPS-Activated RAW264.7 Cells

**DOI:** 10.1155/2019/1018274

**Published:** 2019-05-06

**Authors:** Hui Liao, Yuanping Li, Xiaoru Zhai, Bin Zheng, Linda Banbury, Xiaoyun Zhao, Rongshan Li

**Affiliations:** ^1^Department of Pharmacy, Shanxi Provincial People's Hospital of Shanxi Medical University, Taiyuan 030012, China; ^2^China Institute for Radiation Protection, Taiyuan 030006, China; ^3^NSW Health Pathology, Lismore Base Hospital, Hunter Street, Lismore, 2480 NSW, Australia; ^4^School of Pharmacy, Shanxi Medical University, Taiyuan 030001, China; ^5^Department of Nephrology, Shanxi Provincial People's Hospital of Shanxi Medical University, Shanxi Kidney Disease Institute, No. 29 Shuangtasi Street, Taiyuan, 030012 Shanxi, China

## Abstract

**Objective:**

Safflower has antioxidant and anti-inflammatory activities. The two forms of preparations for safflower which are widely used in China are injection and decoction. The first step of the process for preparing an injection involves extracting safflower with water, which actually yields a decoction. This study is intended to investigate how the preparation process influences the anti-inflammatory activity of safflower *in vitro*.

**Methods:**

Five samples, including a decoction (sample 1) and an injection (sample 5) of safflower, were prepared according to the national standard WS3-B-3825-98-2012 and were analyzed by the oxygen radical absorbance capacity (ORAC) method and the 1,1-diphenyl-2-trinitrophenylhydrazine (DPPH) method for comparison. Sample 1 and sample 5 were further tested by the Griess assay and ELISA for their effects on nitric oxide (NO) production and interleukin- (IL-) 1*β* content in lipopolysaccharide- (LPS-) activated RAW264.7 cells. The protein and mRNA levels of inducible nitric oxide synthase (iNOS) and IL-1*β* were measured by Western blotting and real-time quantitative PCR.

**Results:**

Sample 5 showed a significantly higher ORAC value and a lower half inhibitory concentration (IC_50_) for DPPH scavenging activity as compared to the other four samples (*p* < 0.05). LPS significantly upregulated the mRNA and protein expressions of iNOS and IL-1*β* as compared to the solvent control (*p* < 0.01). As compared to sample 1, sample 5 significantly decreased NO production, iNOS protein expression, and the contents of IL-1*β* mRNA and IL-1*β* protein at both 100 *μ*g/ml and 200 *μ*g/ml (all: *p* < 0.05) and significantly downregulated iNOS mRNA expression at 100 *μ*g/ml (*p* < 0.05).

**Conclusions:**

Results of this study demonstrate that the safflower injection prepared according to the national standard has a significant effect of suppressing protein and mRNA expressions of iNOS and IL-1*β* as compared to its traditional decoction.

## 1. Introduction

Safflower is the tubular flower of *Carthamus tinctorius*. According to theories of Chinese traditional medicine, safflower has effects of promoting blood circulation and removing blood stasis [1]. Modern pharmacological researches and clinical examinations suggest that safflower is a promising agent for ameliorating myocardial ischemia, trauma and pain of joints, etc. [2]. In China, safflower decoction is a traditional preparation, while safflower injection is regarded as a “product of herb's modernization” [3]. A recent article reviewed 956 papers regarding the use of safflower injection in the treatment of a variety of diseases such as cerebral infarction, transient ischemic attack, and chronic glomerulonephritis [4].

The effects of safflower injection have been pharmacologically and clinically proved to be related to the antioxidant and anti-inflammatory activities [5–7]. The protective effect of safflower injection against isoprenaline-induced acute myocardial ischemia in rats is likely to be related to a decreased inflammatory response mediated by tumor necrosis factor alpha (TNF-*α*) and interleukin- (IL-) 6 in the heart tissue [5]. Some clinical researches showed that safflower injection could be used to treat acute lung injury by decreasing TNF-*α* and IL-8 levels as measured in patient's serum [6]. Another clinical study found that the serum levels of IL-6 and IL-10 were significantly elevated in patients with acute cerebral infarction (ACI) and safflower injection exerted certain neuroprotective effects in ACI patients by suppressing IL-6 and IL-10 expressions [7].

Safflower injection has been widely used in China, and the process for preparing a safflower injection starts from the traditional decoction [7]. We were interested in how the process for preparing a safflower injection could influence its antioxidant and anti-inflammatory activities. The process for preparing a safflower injection includes the step of water decoction followed by alcohol precipitation according to the current national standard for injections, “WS3-B-3825-98-2012” (hereinafter referred to as WS3-2012) [8]. Our preliminary work showed that the safflower extract obtained according to WS3-2012 had an antioxidant effect which was associated with the activity of inhibiting nitric oxide (NO) production in lipopolysaccharide- (LPS-) activated RAW264.7 cells [9]. In this paper, five samples obtained during the process were compared in terms of antioxidant activity by the oxygen radical absorbance capacity (ORAC) method and the 1,1-diphenyl-2-trinitrophenylhydrazine (DPPH) radical scavenging method. NO production, IL-1*β* content, inducible nitric oxide synthase (iNOS), and IL-1*β* protein and mRNA expressions in LPS-activated RAW264.7 macrophages were further measured after treatment with the first water decoction sample and the final safflower injection sample.

## 2. Methods

### 2.1. Preparation of Samples

**Figure 1:**
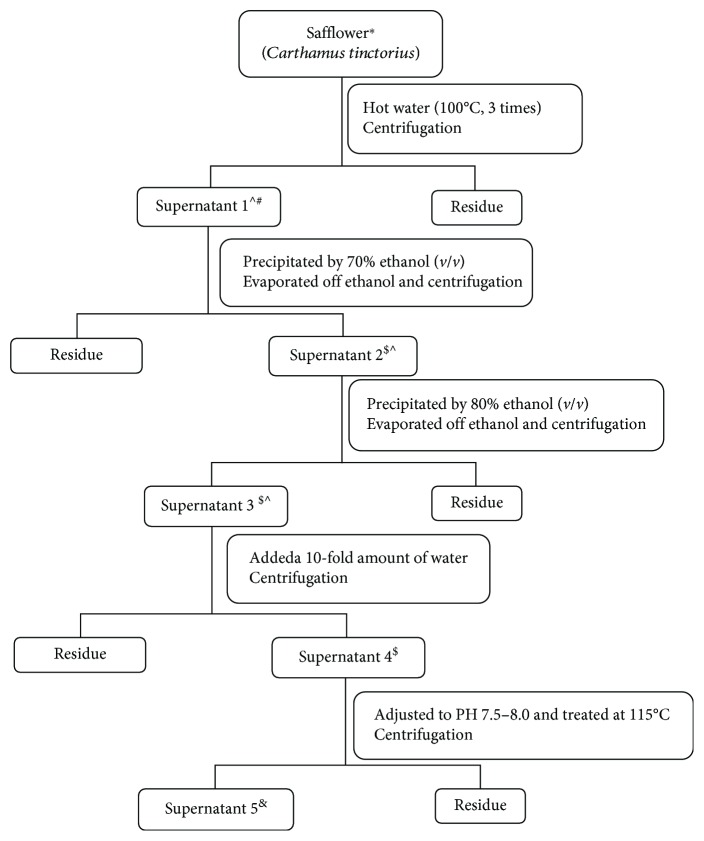
Flowchart on the process for producing safflower injection and the five samples obtained in the research [8]. ^∗^Safflower: the 20 kg dried herb. ^#^Supernatant 1: the water decoction, and 20 ml of it was obtained as sample 1. ^$^20 ml of each of the extracted supernatants 2, 3, and 4 was obtained as sample 2, sample 3, and sample 4, respectively. ^&^Supernatant 5: the 40000 ml safflower injection, and 20 ml was sampled as sample 5. ^The filtrate was concentrated to a relative density of 1.10–1.14 for supernatant 1, 1.16–1.20 for supernatant 2, and 1.02–1.04 for supernatant 3.

Safflower (*Carthamus tinctorius*) was produced in Xinjiang province and met the standard in the Chinese Pharmacopoeia, 2015 [10]. The safflower injection was manufactured by Shanxi Huawei Pharmaceutical Co. Ltd. according to WS3-2012 [8], as was shown in Figure 1.

20 ml of each of the five extracted supernatants shown in Figure 1 was labeled as sample 1 (traditional water decoction), sample 2, sample 3, sample 4, and sample 5 (safflower injection product). 10 ml of each sample was accurately pipetted into a container and dried in vacuo to a constant weight. All liquid and dried samples were stored at 0-4°C for future use. Five liquid samples were subjected to high-performance liquid chromatography (HPLC) profiling, and the dried samples were used to determine the antioxidant activity by the ORAC, DPPH methods, and *in vitro* cell assays.

### 2.2. HPLC Profiling of the Five Samples and Content Analysis of Hydroxysafflor Yellow A (HSYA) in Sample 1 and Sample 5 [8, 10]

In HPLC profiling, octadecylsilane-bonded silica used was a Gemini C18 (250 × 4.6mm, Phenomenex, Torrance, CA, USA) at a column temperature of 25°C. Gradient elution was carried out with acetonitrile as mobile phase A and aqueous trifluoroacetic acid (0.05%) as mobile phase B. The detection wavelength was 223 nm. 10 *μ*l of HSYA (96.5%, China National Institutes for Food and Drug Control, Beijing) control solution and each sample solution were, respectively, injected into the liquid chromatograph column and ran for 70 min [8]. The contents of HSYA in sample 1 and sample 5 were measured with reference to the Chinese Pharmacopoeia, 2015 [10].

### 2.3. Determination of the Antioxidant Activities of the Five Samples by the ORAC Method [11]

#### 2.3.1. Preparation of the Standard Curve

6-hydroxy-2,5,7,8-tetramethyl-2-carboxylic acid (Trolox, 97.0%, Aldrich Corporation, USA), a water-soluble analog of vitamin E, was used as the standard. Firstly, 10 *μ*l of 75 nM 3′,6′-dihydroxy-spiro[isobenzofuran-1[3H],9′[9H]-xanthen]-3-one, also known as fluorescein disodium (FL) (95%, Aldrich Corporation, USA), was added to each well. Then, 20 *μ*l of Trolox at concentrations of 6.25, 12.5, 25, and 50 *μ*M was added in triplicate. Finally, 170 *μ*l of 17 mM 2′-Azobis(2-amidinopropane) dihydrochloride (AAPH) (≥98.0%, Wako Pure Chemical Corporation, USA) was added to each well and the fluorescence change was dynamically recorded on the Wallac Victor 3 fully automated quantitative mapping microplate reader (PerkinElmer, USA) every 1 min for 35 min at 37°C. Trolox was diluted with deionized water, and FL and AAPH were diluted with 75 mM phosphate buffer solution (PBS) (in-house). 20 *μ*l of deionized water was included as a solvent control. The fluorescence-time graph was plotted using the workout program, and the area under the curve was calculated. The following standard curve equation for Trolox was obtained with the area under the curve as the ordinate and the Trolox concentration as the abscissa: *y* = 1.0259*x* + 0.0960, *r* = 0.9959.

#### 2.3.2. Determination of the ORAC Value

*(1) Positive Control Group*. 20 *μ*l of curcumin (>95%, China National Institutes for Food and Drug Control, Beijing, China) was incubated with 10 *μ*l of 75 nM FL and 170 *μ*l of 17 mM AAPH in a total volume of 200 *μ*l. The tested concentrations of curcumin were 1, 2, 4, and 8 *μ*M in triplicate.

*(2) Five Safflower Samples*. Briefly, 20 *μ*l of safflower samples at 25, 50, 100, and 200 *μ*g/ml and 20 *μ*l of HSYA samples at 12.5, 25, 50, and 100 *μ*M were tested. FL and AAPH were added following the same steps as those of curcumin.

*(3) Solvent Control*. A solvent control comprising DMSO for curcumin and a deionized water control for the five samples and HSYA were included.

The ORAC values (in *μ*mol·TE/g) of the positive curcumin, safflower samples, and HSYA were calculated from the linear equation of the Trolox standard.

### 2.4. Determination of the Antioxidant Activities of the Five Samples by the DPPH Method [12]

#### 2.4.1. Preparation of the DPPH Standard Curve

0.5, 1.0, 2.0, 3.0, 4.0, and 5.0 ml of a 50 *μ*g/ml solution of DPPH (>97.0%, Tokyo Chemical Industry Corporation, Tokyo, Japan) in 95% ethanol were accurately pipetted into 5 ml volumetric flasks, to which ethanol was added to the final volume. The mixture was shaken well. The *A* values were measured at 517 nm. The following standard curve equation for DPPH was obtained with the *A* value as the ordinate and the concentration as the abscissa: *y* = 29.1170*x* + 0.0354, *r* = 0.9999.

#### 2.4.2. Determination of Parameters of the Samples

*(1) DPPH-Negative Control*. 1.0 ml of 95% ethanol was added to 2.0 ml of a 50 *μ*g/ml DPPH solution and mixed well. After the mixture was set aside for 30 min in a 28°C water bath, the *A* value at 517 nm was measured as *A*_*D*_.

*(2) Positive Control*. 0.5 ml of the tested curcumin solutions at 1.56, 3.13, 6.25, 12.5, 25, and 50 *μ*g/ml was thoroughly mixed with 0.5 ml of 95% ethanol, and then 2.0 ml of the 50 *μ*g/ml DPPH solution was added to the mixture.

*(3) Five Safflower Samples*. 0.5 ml of the tested safflower samples at 15.6, 31.3, 62.5, 125, 250, and 500 *μ*g/ml was mixed with 0.5 ml of 95% ethanol, and other steps were the same as those for the positive control.

*(4) HSYA Sample*. 0.5 ml of the tested HSYA at 3.2, 6.3, 12.5, 25, 50, and 100 *μ*g/ml was mixed with 0.5 ml of 95% ethanol, and other steps were the same as those for the positive control. All the *A* values of curcumin, five safflower samples, and HSYA were recorded as *A*_*T*_.

*(5) Blank*. The *A* value of 3.0 ml of 95% ethanol was measured as *A*_*B*_.

*(6) Solvent Control*. 0.5 ml of DMSO (a solvent for curcumin) and deionized water (a solvent for safflower samples and HSYA) was mixed with 2.5 ml of 95% ethanol, and their *A* values were measured as *A*_*S*_.

The DPPH scavenging rate of the samples at different concentrations was calculated according to the following equation:

(1) $$\begin{array}{c} \left(1-\frac{A_{T}-A_{S}}{A_{D}-A_{B}}\right)\times 100\%. \end{array}$$

The half inhibitory concentration (IC_50_) for DPPH scavenging of the samples, i.e., the corresponding concentration of the sample solution when the DPPH radical scavenging rate is 50%, was calculated.

### 2.5. Cell Culture

RAW264.7 cells, a mouse macrophage cell line, were purchased from Shanghai Cell Institute (Shanghai, China) and cultured in colorless Dulbecco's modified Eagle's medium (DMEM) supplemented with heat-inactivated fetal bovine serum (10%), D-glucose (3.5 mg/ml), Na pyruvate (100 mM), L-glutamine (2 mM), penicillin (100 U/ml), streptomycin (100 *μ*g/ml), and amphotericin B (250 *μ*g/ml) at 37°C in a 5% CO_2_ incubator.

### 2.6. Determination of NO and IL-1*β* Levels in LPS-Activated RAW264.7 Cells in the Presence of Sample 1 and Sample 5

NO and IL-1*β* levels were determined in RAW264.7 cells (98 *μ*l, plated at 1 × 10^6^ cells/ml). The samples (1 *μ*l each) were added to the cells, which were then stimulated with LPS (1 *μ*l, 0.5 *μ*g/ml, Wako Chemicals USA Inc., Richmond, VA, USA) after 2 h. Nitrite, a stable end product of NO metabolism, was measured using the Griess reaction [13] after another 22 hours, and IL-1*β* was measured using an ELISA kit commercially available from Wuhan Boster Biological Technology (Wuhan, China). All samples and controls were assayed in sextuplicate.

### 2.7. Real-Time Quantitative PCR of iNOS and IL-1*β* in the Presence of Sample 1 and Sample 5 [14, 15]

Total RNAs were extracted from solvent-treated RAW264.7 cells, LPS-activated cells, and sample-treated LPS-activated cells with TRIzol Reagent (Ambion, USA). Equal amounts (1 *μ*g) of RNAs were reverse transcribed using a high-capacity RNA-to-cDNA PCR kit (Takara, Beijing, China). Mouse gene PCR primer sets for iNOS and IL-1*β* were obtained from SABiosciences (Germantown, MD). The Power SYBR Green PCR Master Mix (Applied Biosystems) was used with the step-one-plus real-time PCR system (Applied Biosystems). The protocol included denaturing for 15 min at 95°C, 40 cycles of three-step PCR including denaturing for 15 sec at 95°C, annealing for 30 sec at 58°C, and extension for 30 sec at 72°C, with an additional 15-second detection step at 81°C, followed by a melting profile from 55°C to 95°C at a rate of 0.5°C per 10 sec. The samples of 25 ng cDNA were analyzed in quadruplicate in parallel with RPLP1/3 controls. Standard curves (threshold 1 cycle vs. log 2 pg cDNA) were generated from a series of log dilutions of standard cDNA (reverse transcribed from mRNA from RAW264.7 cells in growth media) from 0.1 pg to 100 ng. Initial quantities of experimental mRNA were then calculated from the standard curves and averaged using the SA Bioscience software. The ratio of the experimental marker gene (iNOS or IL-1*β*) to RPLP1/3 mRNA was calculated and normalized to the solvent control.

### 2.8. Western Blotting of iNOS and IL-1*β* in the Presence of Sample 1 and Sample 5 [16]

The treated cells were removed from the culture media and extracted with the RIPA lysis buffer from Beyotime Biotech (Jiangsu, China) for 30 min. Supernatants were collected after the tubes were centrifuged at 10000 g for 40 min at 4°C. The protein concentrations were determined using a BCA Protein Assay Kit from Wuhan Boster Biological Technology (Wuhan, China). Samples containing 50 *μ*g of protein were resolved by 12% SDS-PAGE electrophoresis and transferred to nitrocellulose membranes (Whatman International Ltd., Maidstone, Germany). Nonspecific binding was blocked by immersing the membranes into 5% nonfat dried milk and 0.1% (*v*/*v*) Tween 20 in PBS for 3 h at room temperature. After rinsing with a washing buffer (0.1% Tween 20 in PBS) several times, the membranes were incubated with a primary antibody against iNOS at 1 : 1000 dilution (catalog no. ab49999, Abcam) or an antibody against IL-1*β* at 1 : 1000 dilution (catalog no. ab150777, Abcam) overnight at 4°C. The membranes were washed several times, then incubated with a corresponding anti-mouse secondary antibody IgG conjugated to HRP (Cell Signaling Technology, Danvers, MA) at room temperature for 3 h, and analyzed by the Quantity One analysis system (Bio-Rad, Hercules, CA, USA). GAPDH at a dilution of 1 : 2000 (catalog no. ab 9483, Abcam) was used as an internal loading control.

### 2.9. Statistical Analysis

The SPSS 19.0 software (IBM, Armonk, NY, US) was used for statistical analysis. All the data were expressed as mean ± standard error of the mean. For continuous variables, comparisons among groups were conducted by one-way analysis of variance followed by Dunnett's multiple comparisons test. All the *p* values reported were two tailed, and *p* < 0.05 was set as the level of significance.

## 3. Results

### 3.1. The HSYA Contents in Sample 1 and Sample 5 and HPLC Profiling Results of the Five Samples

According to WS3-2012, the content of HSYA should be no less than 0.10 mg/ml [8]. The results showed that sample 5 obtained in the present study contained 0.20 ± 0.01 mg/ml of HSYA (*n* = 3), which met the requirements of WS3-2012, and equaled to 11.2 ± 0.2 mg of HSYA per 1 g extract. The content of HSYA was also measured in sample 1, and the result was 43.3 ± 0.8 mg of HSYA per 1 g extract.

**Figure 2:**
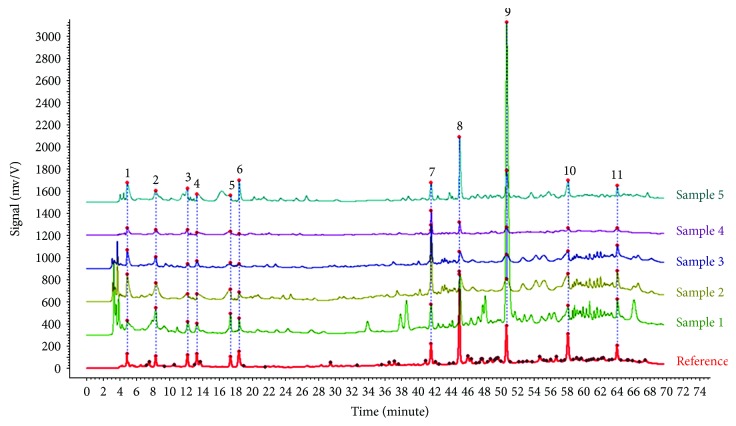
High-performance liquid chromatography profiles of the reference and five samples. Sample 1: the safflower decoction; sample 5: the safflower injection. Performance temperature was 25°C. Mobile phase A was gradient elution with acetonitrile, and mobile phase B was aqueous trifluoroacetic acid. The detection wavelength was 223 nm. 10 *μ*l Hydroxysafflor yellow A (HSYA) was the control solution. Run time was 70 min. According to reference fingerprint, sample 5 showed 11 characteristic absorption peaks and peak 9 was confirmed as HSYA [8].

In addition, WS3-2012 specifies 11 characteristic peaks in the HPLC profile of the injection, in which peak 9 represents HSYA (Figure 2, sample 5). The theoretical number of column plates should be no less than 6000 as calculated from the HSYA peak, and the similarity determination of the 11 peaks between the profile of sample 5 and the reference fingerprint should be no less than 0.85 (Figure 2) [8]. The above HPLC indices of sample 5 all met the WS3-2012's requirements. Figure 2 showed that the 11 characteristic peaks were also present in sample 1.

### 3.2. The ORAC Values of the Five Samples and HSYA

**Figure 3:**
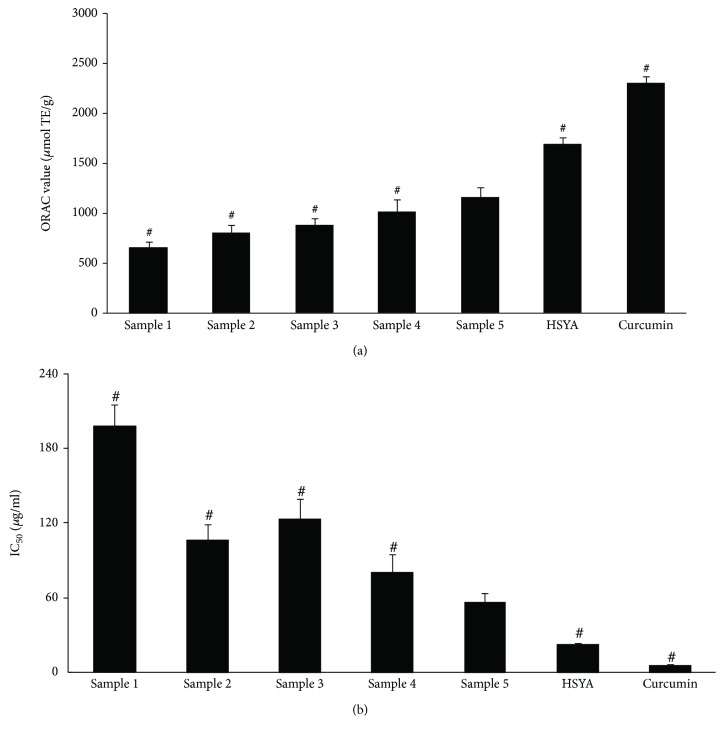
(a) The ORAC values of the samples and HSYA. Sample 1: the safflower decoction; sample 5: the safflower injection. Values are expressed as mean ± standard error (*n* = 6). ^#^*p* < 0.05 versus sample 5. Curcumin was the positive control. ORAC: oxygen radical absorbance capacity; HSYA: Hydroxysafflor yellow A. (b) The IC_50_ values for DPPH scavenging of the samples and HSYA. Sample 1: the safflower decoction; sample 5: the safflower injection. Values are expressed as mean ± standard error of the mean (*n* = 6). ^#^*p* < 0.05 versus sample 5. Curcumin was the positive control. DPPH: 1,1-diphenyl-2-trinitrophenylhydrazine; IC_50_: half inhibitory concentration; HSYA: Hydroxysafflor yellow A.

Sample 1 was prepared by extraction with water.  Figure 3(a) showed that, following several steps of alcohol precipitation and water precipitation, the ORAC value of sample 5 was significantly higher than that of sample 1 (1160 ± 146 *μ*mol · TE/g vs. 650 ± 61 *μ*mol · TE/g; *p* = 0.001) and also higher than those of the other three samples (*p* < 0.05). As an important compound in safflower, HSYA was found to have a significantly higher ORAC value (1702 ± 109 *μ*mol · TE/g) than sample 5 (*p* = 0.001). As a positive control in this study, curcumin exhibited the highest ORAC value (2307 ± 66 *μ*mol · TE/g), which was significantly different from that of sample 5 (*p* < 0.001).

### 3.3. The IC_50_ Value for DPPH Scavenging of the Five Samples and HSYA

Figure 3(b) showed that curcumin, as a reported antioxidant [17], exhibited the lowest IC_50_ value (5.7 ± 1.1 *μ*g/ml), which was most significantly different from that of sample 5 (*p* < 0.001). Among the five samples, sample 5 had the lowest IC_50_ value and sample 1 had the highest IC_50_ value (56.7 ± 7.2 *μ*g/ml vs. 197.6 ± 18.1 *μ*g/ml, *p* < 0.001). The IC_50_ value of HSYA was 23.2 ± 3.4 *μ*g/ml, further confirming its DPPH scavenging activity [12].

### 3.4. Effects of Sample 1 and Sample 5 on NO and IL-1*β* Contents in LPS-Activated RAW264.7 Cells

NO production increased significantly after LPS stimulation as compared to the solvent control (26.8 ± 0.3 *μ*M vs. 6.6 ± 0.1 *μ*M, *p* < 0.001). Also, as compared to that of the solvent control, the IL-1*β* level in the LPS control increased significantly (14.7 ± 0.3 pg/ml vs. 69.4 ± 5.6 pg/ml, *p* = 0.003).

RAW264.7 cells treated with sample 1 and sample 5 at 50, 100, and 200 *μ*g/ml exhibited significantly lower LPS-stimulated NO production than the LPS control (*p* < 0.05). Sample 5 showed a significant inhibitory effect as compared to sample 1 at 100 *μ*g/ml (*p* = 0.020) and at 200 *μ*g/ml (*p* < 0.001).

**Figure 4:**
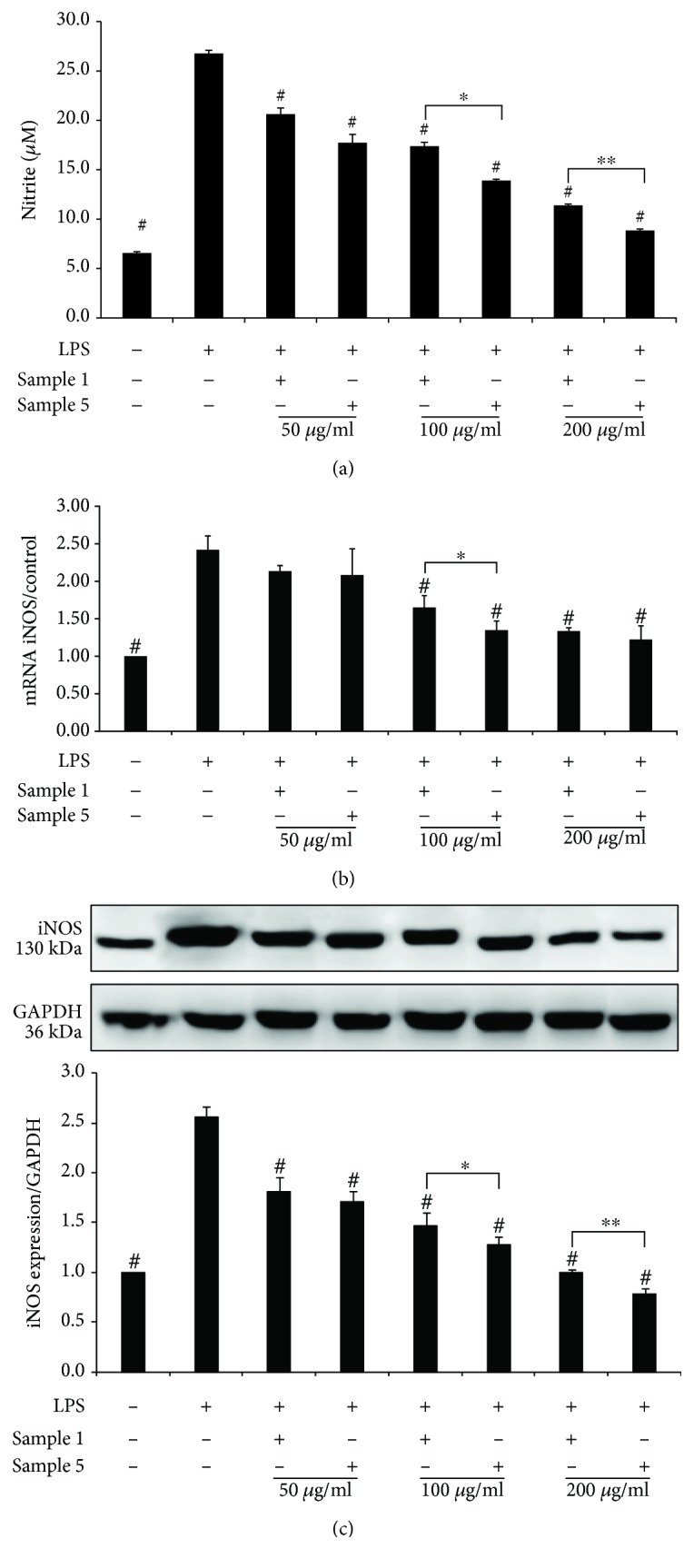
(a) Effects of sample 1 and sample 5 on nitric oxide production in 0.5 *μ*g/ml LPS-activated RAW 264.7 cells. (b) Effects of sample 1 and sample 5 on the mRNA of iNOS in 0.5 *μ*g/ml LPS-activated RAW264.7 cells by real-time quantitative PCR.(c) Effects of sample 1 and sample 5 on the expression of iNOS in 0.5 *μ*g/ml LPS-activated RAW264.7 cells by Western blotting analysis. Sample 1: the safflower decoction; sample 5: the safflower injection. Values are expressed as mean ± standard error of the mean (*n* = 3). ^#^*p* < 0.05 versus LPS control. ^∗^*p* < 0.05 sample 1 versus sample 5 at 100 *μ*g/ml. ^∗∗^*p* < 0.05 sample 1 versus sample 5 at 200 *μ*g/ml. iNOS: inducible nitric oxide synthase; LPS: lipopolysaccharide.

**Figure 5:**
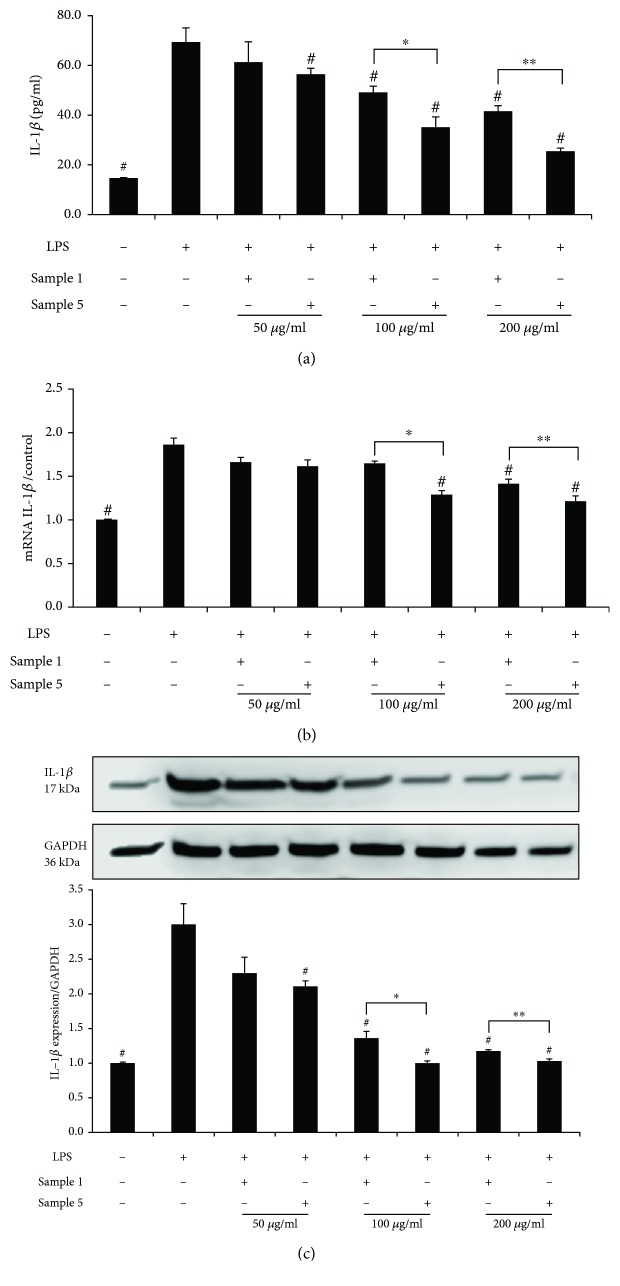
(a) Effects of sample 1 and sample 5 on IL-1*β* in 0.5 *μ*g/ml LPS-activated RAW264.7 cells. (b) Effects of sample 1 and sample 5 on the mRNA of IL-1*β* in 0.5 *μ*g/ml LPS-activated RAW264.7 cells by real-time quantitative PCR. (c) Effects of sample 1 and sample 5 on the expression of IL-1*β* in 0.5 *μ*g/ml LPS-activated RAW264.7 cells by Western blotting analysis. Sample 1: the safflower decoction; sample 5, the safflower injection. Values are expressed as mean ± standard error of the mean (*n* = 3). ^#^*p* < 0.05 versus LPS control. ^∗^*p* < 0.05 sample 1 versus sample 5 at 100 *μ*g/ml. ^∗∗^*p* < 0.05 sample 1 versus sample 5 at 200 *μ*g/ml. LPS: lipopolysaccharide.

Sample 1 at 50 *μ*g/ml did not exhibit a statistically significant inhibitory effect on LPS-stimulated IL-1*β* production (*p* = 0.081 vs. the LPS control). As compared to sample 1, sample 5 showed a significant inhibitory effect on IL-1*β* production at 100 *μ*g/ml (*p* = 0.006) and at 200 *μ*g/ml (*p* = 0.007) (Figures 4(a) and 5(a)).

### 3.5. Effects of Sample 1 and Sample 5 on iNOS and IL-1*β* mRNAs in LPS-Activated RAW264.7 Cells

When compared to those of the solvent control, iNOS mRNA expression increased by approximately 2.42 ± 0.19 fold and IL-1*β* production increased by approximately 1.86 ± 0.08 fold in LPS-activated cells.

When compared to that of the LPS control, iNOS mRNA expression was significantly downregulated by sample 5 at 100 *μ*g/ml (*p* = 0.040) and 200 *μ*g/ml (*p* = 0.019) and sample 1 at 100 and 200 *μ*g/ml (both *p* < 0.01). The significant inhibitory effect on IL-1*β* mRNA was also observed with sample 5 at 100 *μ*g/ml and 200 *μ*g/ml and sample 1 at 200 *μ*g/ml (*p* < 0.05).

Compared to sample 1 at 100 *μ*g/ml, sample 5 at the same concentration significantly decreased iNOS mRNA and IL-1*β* mRNA levels (*p* = 0.013 and *p* = 0.009). Sample 5 at 200 *μ*g/ml also exhibited a similar significant inhibitory effect on IL-1*β* mRNA expression as compared to sample 1 (*p* = 0.011) (Figures 4(b) and 5(b)).

### 3.6. Effects of Sample 1 and Sample 5 on iNOS and IL-1*β* Protein Expressions in LPS-Activated RAW264.7 Cells

Western blotting was used to determine the effects of the decoction sample and injection sample on iNOS and IL-1*β* protein expressions. Figures 4(c) and 5(c) showed that iNOS and IL-1*β* protein expressions increased in LPS-activated RAW264.7cells.

Compared to the LPS control, both sample 1 and sample 5 significantly suppressed iNOS expression at three tested concentrations (*p* < 0.05). Sample 5 suppressed IL-1*β* protein expression at 50, 100, and 200 *μ*g/ml, while sample 1 exhibited a significant suppressing effect on IL-1*β* protein expression at 100 and 200 *μ*g/ml (all: *p* < 0.05).

Sample 1 and sample 5 were significantly different in their abilities of decreasing iNOS protein expression both at 100 *μ*g/ml and at 200 *μ*g/ml (*p* < 0.05). A significant difference in downregulation of IL-1*β* protein expression was also observed between the two groups treated with safflower at 100 *μ*g/ml (*p* = 0.010) and at 200 *μ*g/ml (*p* = 0.002), respectively.

## 4. Discussion

Safflower is well known for its antioxidant effects and has been widely used to treat conditions including musculoskeletal injuries and cardiocerebrovascular diseases [2, 4, 18]. A paper revealed that more than 100 herbal items have been used as topical agents in the treatment of musculoskeletal injuries. In order to verify the efficacies of these herbs, a comprehensive study was proposed, in which five herbs, including safflower, were selected as suitable candidates for further study. The clinical data from the pilot studies confirmed that the effects of safflower were related to its proven antioxidant activities [18].

Traditionally, safflower is clinically used as a water decoction. As a modern preparation [3], the safflower injection prepared by Shanxi Provincial People's Hospital was initially used to treat coronary diseases and cerebral thrombosis in 1973-1974 [19, 20]. Since then, safflower injection has been widely used in the treatment of cardiocerebrovascular diseases [4]. The injection has been studied more extensively than decoction for adverse reactions and the correlation between the antioxidant activity and its active contents [3, 21]. A study entitled “New technology for quality control of traditional Chinese medicine based on active ingredients and its application in safflower injection” was awarded a national prize in 2015. Such efforts have advanced the strategies for quality control and promoted the establishment of a standard system for safflower injection along with the development of relevant industries [22].

Our previous study demonstrated the antioxidant activities of safflower extracts [23]. Another study conducted by our laboratory also showed that safflower injection could decrease NO production in LPS-stimulated RAW264.7 cells [9]. According to WS3-2012, the process for preparing a safflower injection begins with safflower decoction. In this study, 20 kg of safflower was decocted in water three times in a traditional manner: 1 h for the first time, 50 min for the second time, and 30 min for the third time and then subjected to alcohol precipitation twice and recovery with ethanol [8]. We were interested on how the process steps could influence the antioxidant activities of safflower decoction and safflower injection. In this study, five samples obtained from the preparation process (Figure 1) were tested for their antioxidant activities. In recent years, a variety of analytical methods have been used to evaluate the *in vitro* antioxidant capacity of safflower, among which the DPPH method and the ORAC assay were widely used [11, 12].

As a positive control, curcumin displayed the highest ORAC value and lowest IC_50_ value for the DPPH scavenging activity in this study. The anti-inflammatory effect of curcumin is most likely exerted through its ability to inhibit cyclooxygenase-2, lipoxygenase, and iNOS [24]. Our previous research also showed that curcumin, as a positive control, decreased the level of nitrite in LPS-activated macrophages [9]. A review research elucidates that most chronic diseases are closely related to chronic inflammation and oxidative stress and the antioxidant properties of curcumin can play a key role in the prevention and treatment of chronic inflammation diseases [17].

Our results showed that sample 5 exhibited an ORAC value significantly different from other tested samples, in particular, sample 1, as measured by the ORAC and DPPH methods. The IC_50_ values for DPPH scavenging activity of the five samples were also measured, and similar results were observed and shown in Figure 3(b).

HSYA showed a higher ORAC value and DPPH scavenging activity as compared to sample 5 (Figures 3(a) and 3(b)). As a main compound in safflower [8, 12], HSYA showed antioxidant activities *in vivo* and *in vitro*. Some studies aiming at identifying HSYA in the brain tissues of rats suggested that HSYA, which increased the activities of superoxide dismutase and catalase, can be potentially used as a neuroprotective agent for traumatic brain injury [25]. Carthamus yellow, which is composed of safflomin A and safflomin B, provided an anti-inflammatory response by inhibiting the production of NO through downregulating iNOS gene expression in LPS-induced macrophages [26]. HSYA also exerted a protective effect against LPS-induced neurotoxicity in dopaminergic neurons through a mechanism that may be associated with the inhibition of IL-1*β*, TNF-*α*, and NO [27].

It is interesting to observe the inconsistency between the HSYA contents and the antioxidant activities of the samples. The content of HSYA in sample 1 was higher than that in sample 5, but the antioxidant activity of sample 1 was significantly lower than that of sample 5, as measured by the DPPH and ORAC methods. So, the first question is raised: what results will be obtained when other methods are used?

The effects of safflower extracts on LPS-induced expression of proinflammatory cytokines, such as iNOS, IL-1*β*, the nuclear receptor NF-*κ*B, and cyclooxygenase-2 (COX-2), were evaluated recently [28, 29]. Some research showed that methanol extracts of safflower (MES) reduced inflammation by suppressing iNOS and COX-2 expressions in LPS-activated cells. The binding to NF-*κ*B and NF-*κ*B luciferase activity were also significantly diminished by MES [28]. The hepatoprotective effects and mechanisms of an extract of *Salvia miltiorrhiza* and safflower were investigated in C57BL/6J mice. Western blotting revealed that DHI inhibited LPS-induced phosphorylation of I*κ*B*α* and NF-*κ*B p65 [29].

To further provide an insight into the anti-inflammatory effect of safflower, LPS-activated RAW264.7 macrophages were used for further investigating the effects of sample 1 and sample 5 on mRNA and protein expressions of iNOS and IL-1*β*. The results showed that iNOS and IL-1*β* expressions in the LPS-stimulated group were significantly higher than those in the solvent control group. Compared to the LPS control, both sample 1 and sample 5 significantly suppressed iNOS and IL-1*β* expressions at different concentrations. Further comparison of the samples showed that sample 5 exhibited a significantly higher inhibitory effect on protein and mRNA expressions of both iNOS and IL-1*β* than sample 1.

The above results were in accordance with those obtained from the ORAC and DPPH methods, confirming that the current standard process for preparing a safflower injection can ensure a higher antioxidant activity of the final product than the first water decoction. However, the second question is raised: as there were more HPLC peaks and higher HSYA content in sample 1 than in sample 5 (Figure 2), why did sample 5 possess a higher antioxidant activity than sample 1?

Content determination is an important means for evaluating the product quality and explaining the pharmacological results. Only active ingredients in safflower, such as safflower yellow, HSYA, kaempferol, and quercetin, can exert positive roles [30, 31]. We hypothesize that some interfering substances were removed during the preparation process while the antioxidant compounds were retained. A study involving depletion of some active ingredients supports our hypothesis by showing that several main components such as HSYA, dehydrated safflower yellow B, and 6-hydroxykaempferol-3,6-di-*O*-glucoside-7-*O*-glucuronide not only play a direct antioxidant role but also synergize [32]. It will be of particular interest to further study synergistic combinations of the compounds present in safflower injection.

It is also interesting to identify promising candidates in safflower injection that can be used in future immunotherapeutic strategies. Some researches on the compounds in safflower injection have already obtained positive results. Recently, three active constituents in safflower injection, i.e., HSYA, sirongoside, and (8Z)-decaene-4,6-diyne-1-*O*-*β*-D-glucopyranoside, were identified by HPLC [33]. The contents of uridine, guanosine, and adenosine in the injection were also determined by HPLC. Nucleosides, such as uridine, have an effect against platelet aggregation [34]. Sixteen compounds were isolated from safflower injection, including (1) scutellarin, (2) kaempferol-3-*O*-*β*-rutinoside, (3) HSYA, (4) rutin, (5) coumalic acid, (6) adenosine, (7) syringoside, (8) (3E)-4-(4′-hydroxyphenyl)-3-buten-2-one, (9) (8z)-decaene-4,6-diyne-1-*O*-*β*-*D*-glucopyranoside, (10) 4-hydroxybenzaldegyde, (11) (2E, 8E)-tetradecadiene-4,6-diyne-1,12,14-triol-1-*O*-*β*-*D*-glucopyranoside, (12) kaem-pferol-3-*O*-*β*-sophorose, (13) uridine, (14) roseoside, (15) cinnamic acid, and (16) kaempferol. Compounds 1, 2, 7, 9, 11, and 12 were isolated from the safflower injection for the first time. The results indicated that all the tested compounds but compound 5 exhibited potent antioxidant and anti-inflammatory activities, while compounds 2, 3, 9, and 12 showed strong activities against platelet aggregation [35].

All the efforts above help us understand the interaction of multiple components. The change in the proportion of active ingredients caused by the extraction process and the possibility of a synergistic antioxidant activity need to be further studied. It is also necessary to identify all the peaks in Figure 2 and observe how they change during the extraction process. In summary, the present study, for the first time, provides *in vitro* evidence that the “modern” safflower injection significantly suppresses expressions of both iNOS and IL-1*β* in mRNA and protein levels in LPS-activated RAW264.7 cells as compared to the traditional water decoction. The compounds in safflower injection need to be identified before further *in vivo* studies on the molecular mechanism are conducted.

## Data Availability

The data used to support the findings of this study are included within the article.
